# Inadequacy of Protein Intake in Older UK Adults

**DOI:** 10.3390/geriatrics5010006

**Published:** 2020-02-12

**Authors:** Susan Morris, James D. Cater, Mark A. Green, Alexandra M. Johnstone, Jeffrey M. Brunstrom, Emma J. Stevenson, Elizabeth A. Williams, Bernard M. Corfe

**Affiliations:** 1 Molecular Gastroenterology Research Group, Department of Oncology & Metabolism, The Medical School, The University of Sheffield, Sheffield S10 2RX, UK; sue-morris@hotmail.co.uk (S.M.); james.d.cater@googlemail.com (J.D.C.); 2 Department of Geography and Planning, School of Environmental Sciences, University of Liverpool, Liverpool, Liverpool L69 3BX, UK; mark.green@liverpool.ac.uk; 3 The Rowett Institute, School of Medicine, Medical Sciences and Nutrition, University of Aberdeen, Aberdeen AB25 2ZD, UK; alex.johnstone@abdn.ac.uk; 4 Nutrition and Behaviour Unit, School of Psychological Sciences, University of Bristol, UK. & National Institute for Health Research, Bristol Biomedical Research Centre, University Hospitals Bristol NHS Foundation Trust and University of Bristol, Bristol BS8 1TU, UK; Jeff.Brunstrom@bristol.ac.uk; 5 Human Nutrition Research Centre, Population and Health Sciences Institute, Faculty of Medical Sciences, Newcastle University, Newcastle upon Tyne NE2 4HH, UK; emma.stevenson@newcastle.ac.uk; 6 Department of Oncology & Metabolism, The Medical School, The University of Sheffield, Sheffield S10 2RX, UK; e.a.williams@shef.ac.uk; 7 Insigneo Institute for in silico Medicine, The University of Sheffield, Sheffield S10 2RX, UK

**Keywords:** protein, older adults, protein intake, protein requirement, diurnal eating behavior

## Abstract

The current dietary recommendation for protein intake in the UK is 0.75 g/kg/day, however, this population-wide recommendation does not necessarily reflect altered requirements for older adults to maintain muscle protein synthesis, nor does it encompass the potential impact of intake timing. Optimal muscle protein synthesis in older adults requires both higher intake requirements and a distribution of protein intake above a 25 g threshold, three times across the day. This study aimed to describe the protein intake of older adults in a UK region and compare the results to recommendations. The study re-assessed two existing datasets with rich diet information for older adults in the South Yorkshire area. Data were extracted from food diaries of 256 adults aged between 65 and 89 years old (mean ± SD 72.4 ± 5.3 years). Quantity and timing of intake were coded using Nutritics software and compared to recommendations. The relationship between body mass index (BMI), age, and protein intake was explored. Fewer than 50% of the participants met current UK recommendations (0.75 g/kg/day) and fewer than 15% met the ESPEN 1.2 g/kg/day age-specific recommendation. Only one participant met the 25 g/meal recommendation across three meals. These findings suggest that the older adult population is not achieving recommendations to maintain muscle protein synthesis. Nonetheless it identifies several straightforward opportunities for improvement, notably elevation of morning intake.

## 1. Introduction

The population of older adults is increasing worldwide [1]. Ageing is typically associated with a decline in muscle mass and strength that impacts physical function and increases risk of mortality [2]. This decline in muscle mass and strength characterizes sarcopenia and dynapenia, respectively, which can be attributed in part to a disruption between the continuous process of muscle protein synthesis (MPS) and muscle protein breakdown [3]. Age-related anabolic resistance to MPS is believed to increase dietary protein requirements for the initiation of protein synthesis [4] and MPS is further thought to be dependent on dietary protein quality and distribution intake throughout the day [5].

Current UK guidelines for dietary protein recommend a Reference Nutrient Intake (RNI) of 0.75 g of protein per kilogram of body weight per day (g/kg/d) [6]. However, recent research shows higher intakes are more beneficial in the elderly to maintain muscle mass and muscle function (summarized in Table 2 of reference [7]) which has led international bodies to recommend an increased RNI of 1.2 g/kg/d for optimal MPS in healthy older adults [7]. The suggested amount for phased intake is 25 to 30 g of protein, three times a day in older adults with an emphasis on the consumption of leucine-rich proteins (e.g., meat, fish, dairy, some pulses) for the activation of MPS [5].

Current estimates of UK protein intake are principally derived from the National Diet and Nutrition Survey (NDNS) [8]. In order to explore patterns of protein intake in older adults as part of the Protein 4 Life collaboration [9], a secondary analysis of two previous studies (Food and Immunity Trial (FIT) [10] and Novel Assessment of Nutrition and Ageing (NANA) [11]) was undertaken to assess both quantity and timing of protein intake in a UK regional population.

## 2. Materials and Methods

Secondary analysis was undertaken of food diaries and linked metadata on sex, age, and BMI previously collected in the “Food and Immunity Trial” (FIT) and “Novel Assessment of Nutrition and Ageing” (NANA), respectively, which were medium-scale studies in older adults which included collection of food diaries across an intervention (FIT) and as a reference for a digital tool (NANA) projects (reported elsewhere [10] and [11], ethical approval stated in each respective paper). Food diaries were available from 120 men and 136 women, between the ages of 65 and 89 years (mean 72.4 ± 5.3 yr) living independently. One weekday of each four-day food diary was used in this analysis and intake data and timing were analysed using Nutritics software package v4.315. In order to assess distribution of protein intake across the day, dietary intake was calculated for each of four intervals, 5 a.m. to 11 a.m., 11 a.m. to 5 p.m., 5 p.m. to 11 p.m., and 11 p.m. to 5 a.m. These intervals were chosen to span mealtimes across the three main daily meals, whilst acknowledging individual differences, to assess per-meal protein intakes. Further analysis of the data calculated protein intake as g/kg/d to assess whether this regional population (i) met the daily total UK protein RNI for all adults and (ii) distributed protein intake across the day to achieve optimal muscle maintenance [5]. Protein intake according to BMI and age was also considered using correlation analysis. Data were managed and presented using Microsoft Excel.

## 3. Results

**Figure 1:**
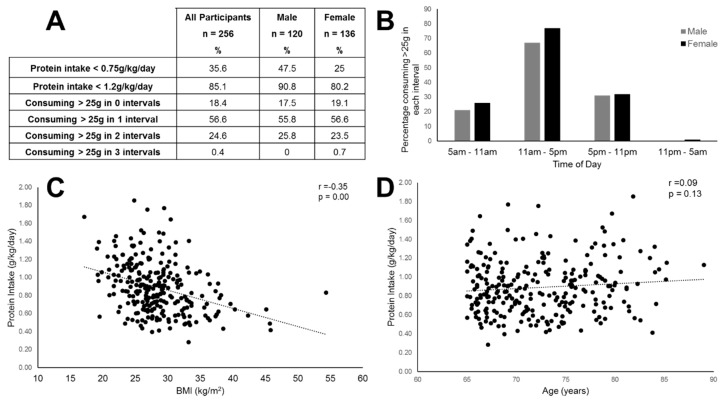
Patterns of protein intake in older adults. (**A**) Summarizes the proportion of the sample achieving the current Reference Nutrient Intake RNI for protein, meeting the higher recommended level, and the proportions consuming adequate per-sitting intakes across the day; (**B**) shows the distribution of intake across the day, presented as percentage reaching 25 g intake in any given window; (**C**) shows the association between body mass index (BMI) and protein intake is moderate and negative (r = −0.35 and *p* < 0.001); (**D**) shows no association between age and daily protein intake in this population (r = 0.09 and *p* = 0.13).

Firstly, the proportion of participants reaching RNI for intake was assessed. Thirty-six percent of individuals consumed lower than 0.75 g/kg/d of protein, with the proportion of males not meeting guidelines exceeding the proportion of females (47.5% vs. 25%). Principle sources of protein were meat, fish, and dairy with around 86% of intake from these leucine-rich sources. When the European Society for Parenteral and Enteral Nutrition (ESPEN) guidelines for older adults were considered, less than 15% participants reached intake levels of 1.2 g/kg/d (Figure 1A). The distribution of protein intake across the day in males and females is presented in Figure 1B. The proportion of participants reaching the recommended 25 g protein/meal is shown for men and women as follows: 67% of males and 77% of females met the 25 g target in the middle of the day, but the proportions were lower for males and females, in the evenings, (31% and 32%), and in the morning, (21% and 26%), respectively. Only one individual met the 25 g/meal target in all three meals (Figure 1A).

The sample had an average body mass index (BMI) of 28.3 kg/m^2^ and 29.7% were obese. There was a negative correlation between BMI and protein intake (r = −0.35 and *p* < 0.01) (Figure 1C). There was no correlation between age and protein intake.

## 4. Discussion

This study found that 35% of participants failed to consume the UK RNI adequate protein, suggesting the South Yorkshire population intake of protein is considerably lower than other populations; a meta-analysis of European elderly populations indicated 10% fail to consume enough protein [12]. With respect to the ESPEN recommended protein intake [7], 85% of the population failed to consume the 1.2 g/kg/day guideline (higher value than the 75% identified in other European countries [13]). With respect to the recommended phased intake of 25 g of protein per meal [5], evidence from this sample indicated that only 24.6% achieved protein intakes over 25 g/sitting twice a day, and only one person out of those sampled managed it thrice daily.

Inadequate protein intake coupled with elevated BMI contributes to the risk of sarcopenic obesity, a double burden on older adults, in turn causing an increased risk of cardiovascular related illness, decreased muscle functionality, and risk of frailty and falls. Accumulation of abdominal adipose tissue masks declining muscle mass, ensuing a vicious cycle of decline, with older adults becoming concomitantly more obese, yet weaker, unable to exercise or even move freely to shop and prepare food [14]. Although body composition data were not available retrospectively, this sample was characterized by higher than ideal BMI, reflecting features and public health concerns around sarcopenic obesity.

The data for this study was collated from food diaries which have well documented limitations, for example, portion size, recall, and reporter bias. Inclusion criteria for one of the source datasets specifically included low fruit and vegetable intake, which may have influenced the intake as their eating patterns were possibly skewed or some patterns counterselected. Additionally, some authors [15] argue the case of correcting for lean body mass to express protein consumed, however, this was not possible with the retrospective analysis undertaken in this study. Only one day’s data from each individual was assessed. As pulsing/regular intake is thought to benefit maintained MPS [5], a weekday was selected from each individual’s record to avoid skewed data from elevated weekend intakes, yielding a substantive number of individuals (256) but limiting the scope to assess intra-individual variation. Notwithstanding these limitations, the intake patterns observed correlate with food habits of older adults in the UK in other research [8].

This study provides detailed information on the protein intake of older adults in a regional population, particularly in relation to timing, identifying opportunities where protein intake could be increased to ensure MPS is optimally stimulated. The reference values of 1.2 g/kg and 25 g phased intake are evidence-based but generic values. In practice, an older population is heterogeneous in its health and illness status, physical activity levels, socioeconomic status, and age range (spanning over 40 years). Using a single guideline for optimal protein intake for the entire population could be inappropriate and research such as ours could help to inform strategies for elevating protein intake at different life stages [4].

## 5. Conclusions

The data suggest that study population fell short of both UK national and European guidelines for protein intake, both in terms of overall intake and diurnal distribution of intake.
